# Segmentation of Tissues and Proliferating Cells in Light-Sheet Microscopy Images of Mouse Embryos Using Convolutional Neural Networks

**DOI:** 10.1109/access.2022.3210542

**Published:** 2022-09-28

**Authors:** LUCAS D. LO VERCIO, REBECCA M. GREEN, SAMUEL ROBERTSON, SIENNA GUO, ANDREAS DAUTER, MARTA MARCHINI, MARTA VIDAL-GARCíA, XIANG ZHAO, ANANDITA MAHIKA, RALPH S. MARCUCIO, BENEDIKT HALLGRíMSSON, NILS D. FORKERT

**Affiliations:** 1 Department of Cell Biology and Anatomy, Cumming School of Medicine, University of Calgary, Calgary, AB T2N 1N4, Canada; 2 Alberta Children’s Hospital Research Institute, University of Calgary, Calgary, AB T2N 1N4, Canada; 3 McCaig Institute for Bone and Joint Health, University of Calgary, Calgary, AB T2N 1N4, Canada; 4 Hotchkiss Brain Institute, University of Calgary, Calgary, AB T2N 1N4, Canada; 5 Department of Radiology, Cumming School of Medicine, University of Calgary, Calgary, AB T2N 1N4, Canada; 6 Department of Orthopaedic Surgery, University of California San Francisco, San Francisco, CA 94115, USA

**Keywords:** Convolutional neural networks, developmental biology, image segmentation, light-sheet microscopy, mouse embryo

## Abstract

A variety of genetic mutations affect cell proliferation during organism development, leading to structural birth defects. However, the mechanisms by which these alterations influence the development of the face remain unclear. Cell proliferation and its relation to shape variation can be studied using Light-Sheet Microscopy (LSM) imaging across a range of developmental time points using mouse models. The aim of this work was to develop and evaluate accurate automatic methods based on convolutional neural networks (CNNs) for: (i) tissue segmentation (neural ectoderm and mesenchyme), (ii) cell segmentation in nuclear-stained images, and (iii) segmentation of proliferating cells in phospho-Histone H3 (pHH3)-stained LSM images of mouse embryos. For training and evaluation of the CNN models, 155 to 176 slices from 10 mouse embryo LSM images with corresponding manual segmentations were available depending on the segmentation task. Three U-net CNN models were trained optimizing their loss functions, among other hyper-parameters, depending on the segmentation task. The tissue segmentation achieved a macro-average F-score of 0.84, whereas the inter-observer value was 0.89. The cell segmentation achieved a Dice score of 0.57 and 0.56 for nuclear-stained and pHH3-stained images, respectively, whereas the corresponding inter-observer Dice scores were 0.39 and 0.45, respectively. The proposed pipeline using the U-net CNN architecture can accelerate LSM image analysis and together with the annotated datasets can serve as a reference for comparison of more advanced LSM image segmentation methods in future.

## I. INTRODUCTION

Structural birth defects and congenital anomalies are a major human health issue, accounting for 300,000 deaths worldwide each year and a significant proportion of the global burden of disease [1]. Most congenital anomalies have a genetic cause that results in a disease by perturbing cellular or molecular processes during embryonic growth and development. Interventions aimed at treating or preventing such diseases require a mechanistic understanding of these disruptions. Many publications report small changes in cell proliferation or apoptosis in response to a perturbation in a model system. However, these changes are often measured in a few stained tissue regions [2], [3], [4]. A major limitation of this approach is that it is difficult to investigate changes across a whole region of tissue or how these changes contribute to the overall anatomical development.

During embryogenesis, tissue layers (ectoderm, mesoderm, and endoderm, along with neural crest cells) form all organs and structures of the body. Each tissue layer has different properties and specific cell fates. For example, the ectoderm gives rise to the neural crest, and during early development, the neural crest is actively migrating and proliferating in response to cues from the ectoderm. The neural crest mixes with the mesoderm to form a tissue type commonly known as mesenchyme. It forms the bones and muscles of the face, which is a common site of structural birth defects. Proliferation and migration of the mesenchyme are thought to be the primary drivers of facial morphogenesis, which can be studied using mouse models [5], [6].

For the study of embryogenesis in mice, fluorescence imaging allows investigating certain biological processes by specifically displaying the molecules and structures involved [7], [8]. However, it is most commonly performed on sections. Once the tissue has been sectioned, it is difficult to analyze the 3D structure and how stains within individual cells relate back to the original morphology. Light-Sheet Microscopy (LSM), a relatively new fluorescence imaging technique, allows 3D imaging of whole biological samples at early developmental stages with a spatial resolution that can display single cells [7]. Furthermore, LSM imaging can be acquired without physical slicing of the samples, thus, preserving the shape of the sample. Technically, LSM illuminates a plane in the sample using a determined frequency, and the fluorescence is imaged using an sCMOS camera perpendicular to the plane. The stack of high resolution 2D images is obtained by moving the illumination plane along the sample. Its 3D resolution depends on the numerical aperture of the detection objective and the intensity profile of the light-sheet, typically presenting a higher in-slice resolution than inter-slice resolution [9], [10], [11]. LSM allows imaging of individual cell nuclei that have been stained for various markers. This work is specifically focused on using DAPI (4’,6-diamidino-2-phenylindole) to stain all nuclei, and pHH3 (phospho-Histone H3 -phospho-serine 28) that only stains actively dividing or proliferating cells.

LSM imaging of mouse embryos has practical drawbacks for its analysis. First, the images are often noisy due to optical aberrations. Moreover, the size of a multi-channel image at 5x zoom can easily approach 300 GB and include thousands of 2D images. These images are also prone to noise and loss of signal due to sample preparation variability and imaging artifacts, particularly in large mammalian samples. Thus, due to the large number of images acquired by LSM, the large size of these images, and the different artifacts that can be present, large-scale manual analysis of LSM images is not feasible. However, to the best of the authors knowledge, there is no publicly available segmentation method so far that was specifically designed for LSM images.

Thus, the aim of this work is to develop and evaluate an automatic framework for segmentation of the mouse embryo anatomy, its tissues, cell nuclei, and proliferating cells that is robust enough to detect differences between individual embryos. The ability to relate cell level changes to morphology in an individual embryo allows analysis of how the two aspects are related. Analysing this individual level relationship will facilitate a new understanding of both normal and abnormal embryonic growth.

### A. RELATED WORK

In recent years, a variety of automatic methods have been applied to fluorescence microscopy, and particularly LSM, for quality improvement and analysis. The high variance of intensities across and between samples in fluorescence microscopy complicates the development of robust methods for automatic image analysis. This variation is a result, for example, of sample preparation, such as excessive non-uniform tissue clearing and/or antibody penetration, noise, loss of signal as the light-sheet travels through the specimen, and due to the non-specific fluorescent signal (background). To date, multiple mechanical and computational methods have been proposed to correct for these problems. Generally, mechanical methods aim to improve the LSM acquisition technique. For example, Turaga and Holy [12] proposed a method to correct for defocus aberrations by tilting the angle of the light-sheet microscopy by a few degrees, whereas the remaining aberrations can be corrected using adaptive optics. Bourgenot *et al*. [13] examined how aberrations can occur in single plane illumination microscopy (SPIM) of zebrafish samples, and proposed a wavefront correction method for this artifact. Among the computational methods for pre- and post-processing, Yang *et al*. [14] developed a method based on Convolutional Neural Networks (CNNs) to automatically assess the focus level quality in microscopy images. Yayon *et al*. [15] proposed a semi-automatic method to normalize images, taking into account the background intensity and signal elements whereas Weigert *et al*. [16] proposed a normalization method based on percentiles to overcome the problem of extreme signal values.

Once the image correction is completed, the image quantification process (e.g., tissue segmentation and cell counting) can be performed with improved accuracy. Particularly for cell segmentation in fluorescence images, Ilastik [17] is a widely used software tool. It provides a trainable pixel segmentation tool, where the user specifies the image features to be extracted and can configure the hyper-parameters of the random forest classifier used in the background. Different techniques for the different tasks based on traditional segmentation methods can be found in the literature. For example, Padfield *et al*. [18] used level-sets and fast marching methods for eukaryotic cell tracking. Salvi *et al*. [19] proposed a watershed-based method for cell segmentation in human-derived cardiospheres, whereas Gertych *et al*. [20] used the watershed method with morphological operators for segmenting carcinoma cells labelled with DAPI. Salvi *et al*. [21] used maximum intensity projection and morphological operators for segmentation of neural cells. An extensive review of automatic cell detection and segmentation methods in a variety of microscopy images was provided by Xing and Yang [22]. Additionally, a variety of methods based on CNNs have been proposed in recent years. For example, Ho *et al*. [23] proposed a 3D CNN model for segmenting nuclei in rat kidneys labelled with Hoechst 33342. The authors specifically highlighted the large amount of annotated data required to train CNNs. To overcome this problem, they trained the CNN with synthetic data and tested it in real images. Falk *et al*. [24] segmented a variety of microscopy images using a generic 2D CNN for segmentation, called U-net. This CNN model was trained using datasets provided by the ISBI Cell Tracking Challenge 2015 [25] and by applying data augmentation techniques to overcome the problem of limited data for CNN training. McQuin *et al*. [26] used U-nets trained for specific cell segmentation tasks, such as for U2OS cells, which were implemented in the software CellProfiler 3.0. The authors specifically highlighted the possibility of generalizing their models for other related tasks in the future. Stringer *et al*. [27] modified the traditional U-net architecture for their tool called Cellpose, and trained it with microscopic images from different sources, improving its generalization capabilities. However, to the best of our knowledge, there is no comprehensive, publicly available software solution capable of segmenting different tissue and cell types in LSM datasets of mouse embryos. Likewise, we are not aware of annotated datasets to train these kind of segmentation methods. In comparison with mouse brains [21], [28] or zebrafish [9], among others, fluorescence microscopy of mouse embryos presents different challenges related to their large size and intrinsic opacity [29]. Furthermore, the scanning of full samples using LSM presents both unique challenges and opportunities when compared to more conventional methods such as confocal microscopy on sections. LSM avoids the artifacts due to sectioning, but generates artifacts in cell shape due to optical refraction differences. These artifacts in the LSM scan can vary across an individual specimen, causing difficulty for many image processing algorithms [30].

### B. CONTRIBUTION OF THIS WORK

In recent years, U-nets have been successfully used for many medical image segmentation tasks [31]. The original U-net architecture was proposed to automatically segment neuronal structures on 2D electron microscopy images [32]. Since then, this CNN model has been extended to segment other types of structures and data, such as the prostate [33] and ischemic strokes [34] in 3D MRI volumes. In this work, U-nets are used to solve segmentation problems in LSM images of mouse embryos, which present unique challenges. Due to the high in-slice resolution of the images, a 2D approach is proposed in this work, where each image in the z-stack is independently segmented, and the resulting segmentations are combined to obtain a 3D model of the embryo.

The segmentation of mesenchyme and neural ectoderm tissues is a key first step to properly analyse the shapes of these rapidly growing regions at different developmental stages. It also allows a more robust registration of images from different samples acquired at the same age and across different ages. After registration, shape changes can be studied using, for example, geometric morphometrics [35]. In this work, a U-net model is trained and evaluated using a novel database of DAPI-stained LSM images of mouse embryos with corresponding manual segmentations.

The second key aim is to quantify cell proliferation in the mesenchyme to support basic science and clinical research investigating how mitosis and migration drive morphological changes. For nuclei as well as proliferating cells, the cell segmentation U-net model described by Falk *et al*. [24] is re-trained using novel LSM datasets of mouse embryos. More precisely, this U-net model was trained using datasets of DAPI-stained images with corresponding annotated cells for nuclei segmentation. For segmentation of proliferating cells, the U-net was trained using pHH3-stained images with corresponding manual segmentations. Finally, the three segmentation results are combined to create maps of relative proliferation in the mesenchyme.

Thus, the main contributions of this work can be summarized as follows:

- We trained: three CNN models using the widely used U-net architecture for (1) tissue segmentation, (2) cell segmentation in nuclear-stained images, and (3) segmentation of proliferating cells in phospho-Histone H3 (pHH3)-stained LSM images. The CNN models are freely available and can be used to accelerate LSM image analysis. Furthermore, they can serve as a baseline comparison method for more advanced segmentation methods in future.
- The three CNN models were evaluated in detail using a comparatively large number of datasets segmented by two observers, which facilitates computation of the inter-observer agreement for benchmarking. The results clearly demonstrate that simple off-the-shelf tools do not produce suitable results for further analyses and are significantly outperformed by the CNN models developed in this work.
- The datasets and corresponding manual segmentations are also publicly available, which allows other researchers to develop novel segmentation models while training and testing on the same datasets as used for this work, again facilitating direct benchmarking and contributing to open science.

The source code, software, instructional videos, and the novel annotated datasets have been made publicly available at https://github.com/lucaslovercio/LSMprocessing.

## II. MATERIALS AND METHODS

### A. IMAGE ACQUISITION

Five E9.5 and five E10.5 mouse embryos were harvested and fixed overnight in 4% paraformaldehyde. After fixation, they were processed for clearing and staining. The clearing process followed the CUBIC protocol [36]. Briefly described, embryos were incubated overnight in Cubic 1/H20 at room temperature followed by incubation in Cubic 1 at 37°C degrees until clear (1–3 days). Samples were blocked in 5% goat serum, 5% dimethyl sulfloxide, 0.1% sodium azide, and phosphate buffered saline at 37°C for 1.5 days. After this, they were incubated in Anti-Histone H3 (phosphorylated-serine 28) primary antibody (Abcam ab10543) (1:250) and DAPI (1:4000) in 1% dimethyl sulfloxide, 1% goat serum, 0.1% sodium azide, and phosphate-buffered saline (PBS) for 7 days at 37°C with shaking. The target protein of this primary antibody is phosphorylated Histone H3.1 (pHH3), which is expressed during the S phase, decreasing as cell division slows down during the differentiation process. Several washes in PBS were performed for 3 days at room temperature. Next, the samples were incubated with secondary antibody (1:500) (Abcam ab150167) for 5 days at 37°C with gentle shaking. The samples were then washed for several days in PBS at room temperature and were embedded in 1.5% low melt agarose, incubated in 30% sucrose for 1 hour, and then placed in Cubic 2 overnight before imaging. The samples were imaged using a Lightsheet Z1 scanner (Zeiss). Images were acquired using single side illumination at 5x zoom using a minimum of 3 laser channels: 405- DAPI, 488- background, and 647- pHH3. All animal work was approved by the University of Calgary Animal Care and Use Committee (approval number ACC-180040).

### B. DATASETS

**FIGURE 1.**
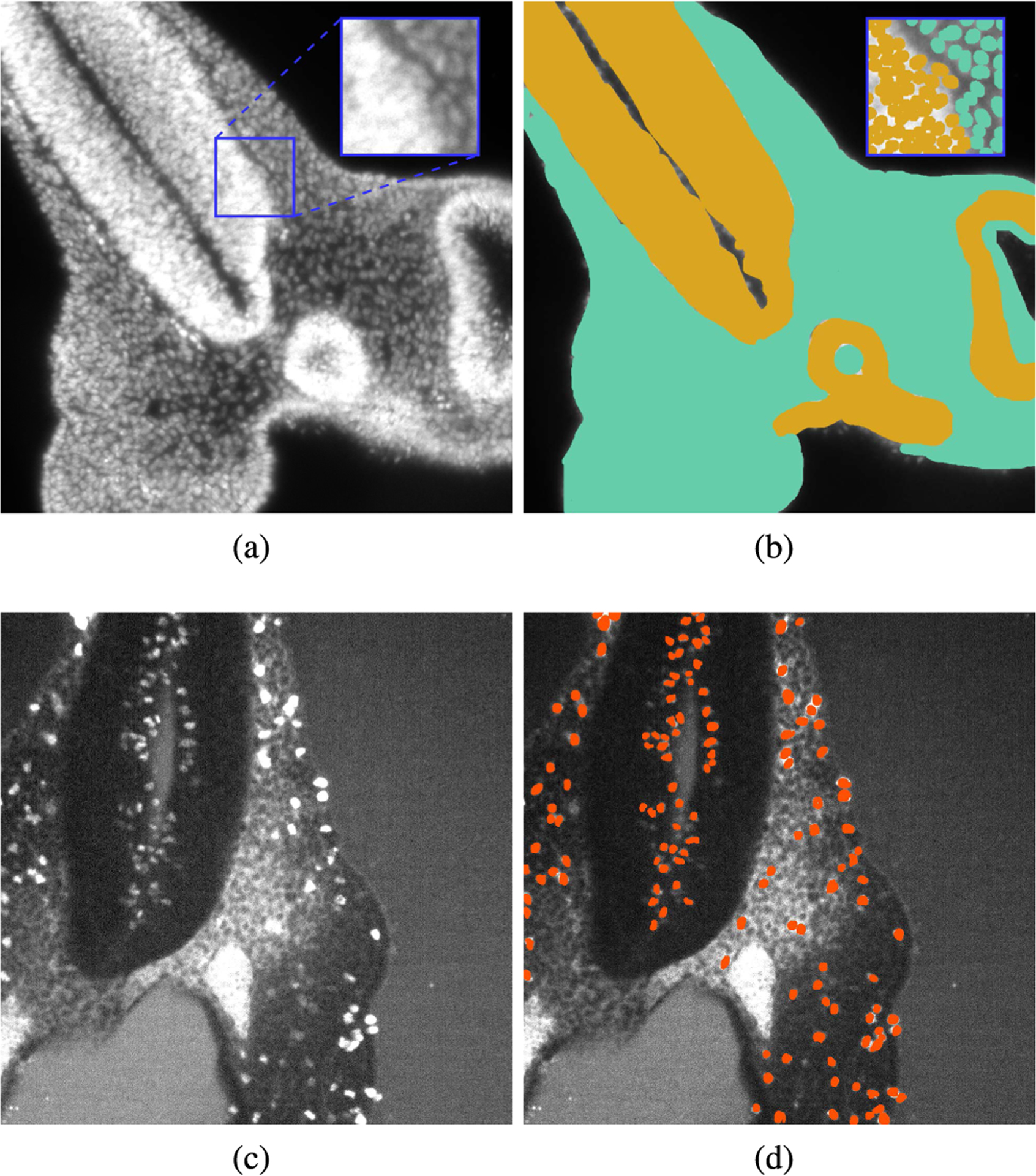
Datasets used for model development and evaluation. (a) Cropped and normalized DAPI-stained image for mesenchyme and neural ectoderm segmentation (DAPI-Tissue). In blue, sub-image used for cell segmentation (DAPI-Cells). (b) Manual segmentation of mesenchyme (aquamarine) and neural ectoderm (yellow) of (a). (c) Cropped and normalized pHH3-stained image (PHH3-Cells). (d) Manual segmentation of proliferating cells marked (orange).

From the DAPI-stained scans of the ten available mouse embryos, random images were selected from the z-stacks for manual tissue segmentation. More precisely, 86 were used for CNN training, 36 for validation, and 54 for testing, following a typical ratio of 50-20-30% for machine learning datasets (DAPI-Tissue dataset, Table 1) [37]. The images were cropped to 1024 × 1024 because the size of the images varies between the scans, depending on the size of the embryo and its position at the time of scanning (Fig. 1a). This patch size was determined to be feasible for human annotation, as the observers required up to five minutes for segmenting each image. Fluorescence microscopy images often present pixels with high intensity variability between scans due to irregular staining, clearing quality, and laser configuration. Thus, prior to further processing and manual annotation, the images were intensity-normalized using percentile-based equalization between the 2^*nd*^ and 99.9^*th*^ percentile [16]. Five expert observers manually segmented the mesenchyme and neural ectoderm tissues in disjoint subsets of the DAPI-Tissue set (Fig. 1b). Each image used for testing was independently segmented by two different observers to assess the inter-observer variability.

For development of the cell segmentation model, a random sub-image with a size of 131 × 131 was extracted for manual cell segmentation from each image in the DAPI-Tissue set (Fig. 1a). Here, the observers segmented single cells, while differentiating them according to the tissue (mesenchyme or neural ectoderm) that they belong to (Fig. 1b). Heavily blurred patches, where single cells could not be separated, were removed from the dataset, resulting in a total of 168 images, whereas 96 were used for training, 17 for validation, and 55 for testing (DAPI-Cells dataset, Table 1). The latter subset was independently segmented by two different observers for variability assessment. Since not all patches contained both types of cells, the proportion of images for the training set was increased from the typical 50% to 57%.

Finally, for the development and evaluation of the CNN model for segmentation of proliferating cells (PHH3-Cells), 155 images were randomly selected from the pHH3 scans of the ten embryos, whereas 86 images were used for training, 20 for validation, and 49 for testing. Each image was intensity-normalized using percentile-based equalization, and a random sub-image of size 1024 × 1024 was extracted [16] (Fig. 1c). In this case, the observers were tasked to segment only proliferating cells (Fig. 1d). The patch size used for this task was different from the images used for cell segmentation in the DAPI-stained scans because proliferating cells represent only a small fraction of the total number of cells. Thus, a human observer can segment a larger image in a similar time frame. Similarly to the previous two subsets, these test images were independently segmented by two different observers for variability assessment.

### C. LSM SEGMENTATION WORKFLOW

**FIGURE 2.**
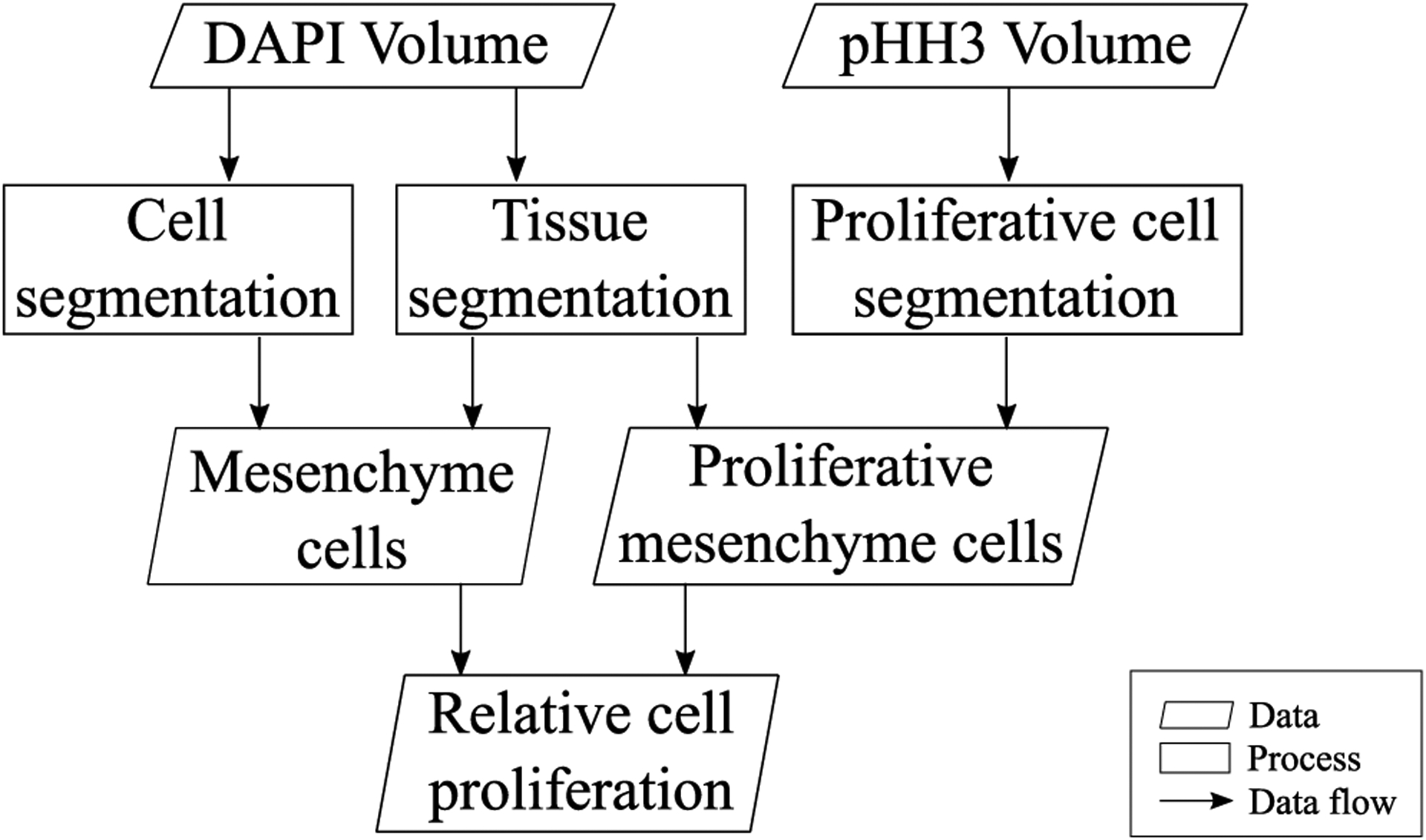
Overview of the proposed light-sheet microscopy segmentation workflow.

Fig. 2 shows the proposed workflow to generate the map of proliferating cells in the mesenchyme of an embryo. The two inputs are the channels belonging to DAPI and pHH3 in light-sheet microscopy scans, and the two main outputs are the segmented mesenchyme and neural ectoderm tissues and the relative proliferating cell volume for the mesenchyme. The methods used for tissue segmentation, cell segmentation, and proliferating cell segmentation are detailed in the following sections.

### D. TISSUE SEGMENTATION

**FIGURE 3.**
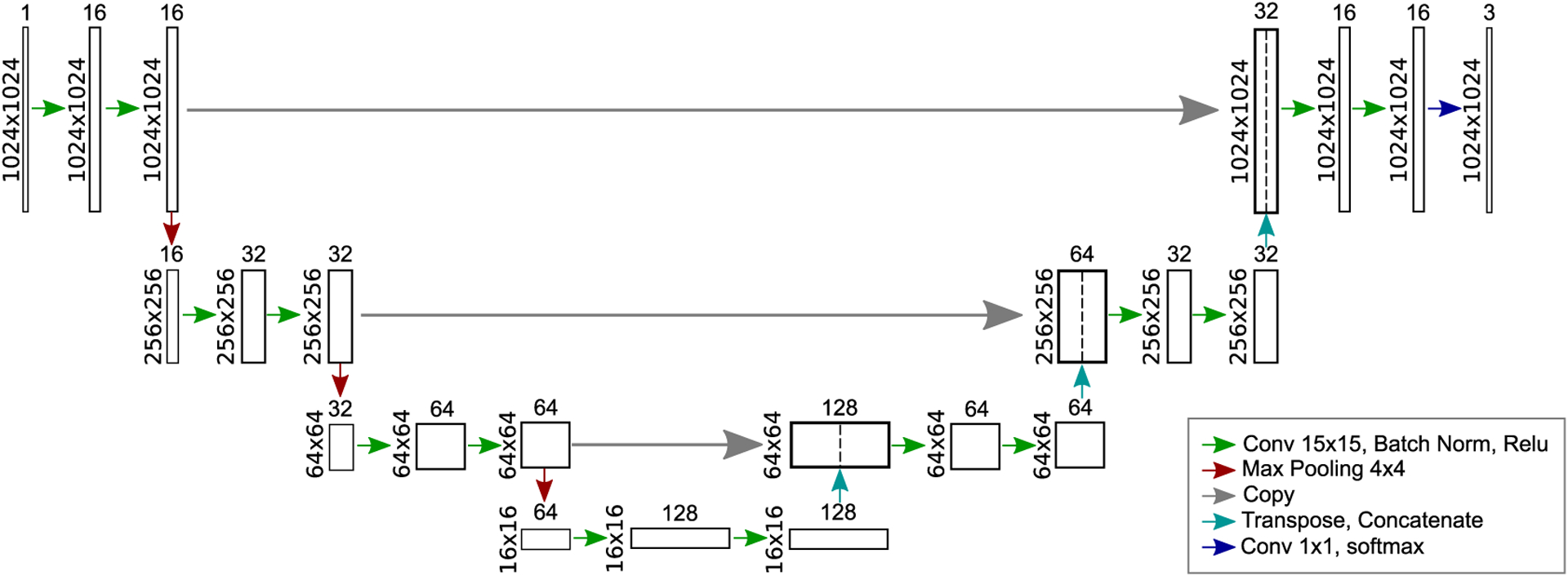
Proposed U-net model architecture for mesenchyme and neural ectoderm segmentation in DAPI-stained images.

For the task of segmenting the mesenchyme and neural ectoderm in DAPI-stained images, and distinguishing these tissues from the background, a simple U-net CNN architecture was used (Fig. 3). A large random search was conducted over various settings of hyper-parameters using the DAPI-Tissue training and validation sets. The hyper-parameters investigated included the batch size, learning rate, number of filters, kernel size, and optimizer method. The search began with a high-dimensional space containing a wide range of hyper-parameters and a wide range of possible settings for each hyper-parameter. At each stage, a large number of models with randomly selected hyper-parameter configurations (within predefined ranges) were trained, and the settings of the best models were recorded. From a manual examination of the settings with the best results, the ranges of possible hyper-parameters were refined after each stage. After the search space was reduced to a feasible size, a grid search was conducted over the remaining possible hyper-parameter values. Finally, the best model was trained and validated five times to ensure that its performance was not a result to random factors inherent in the training process such as the initialization of the weights. Using this procedure, the best model configuration identified used a batch size of 8, an equally weighted combined loss function of soft-Dice and categorical cross-entropy, batch normalization, and ReLU activations in the hidden layers [38], [39] (Fig. 3). This model was trained from scratch, using the default weight initialization from Keras, and the RMSprop optimizer with a learning rate of 1E-4 for 200 iterations. To avoid overfitting, training data augmentation based on deformations (flipping, affine deformations) and based on intensities (blurring, brightness, contrast) was performed on the fly [24], [40]. Furthermore, two dropout layers were included at the end of the contracting path.

### E. CELL SEGMENTATION

U-nets have been previously used for automatic cell segmentation in various imaging modalities [23], [24], [41]. However, one of the major drawbacks identified in previous studies is the lack of annotated datasets to successfully train U-nets for the different microscopy imaging techniques available [24], [25]. A main contribution of this work is the establishment of two datasets (DAPI-Cells and PHH3-Cells) that facilitate the training of CNN models to segment cells in LSM images from whole mouse embryos.

Owing to the limited availability of microscopy datasets, Falk *et al*. [24] trained and evaluated a U-net model for segmenting cells by combining images acquired using different microscopy technologies. Based on this, they developed an ImageJ-Fiji plugin that allows the re-training of this pre-trained U-net. Although the plugin facilitates transfer learning, it was found experimentally that re-training of the pre-trained network had no performance advantage for segmenting DAPI-Cells and PHH3-Cells when compared to randomly initializing the model weights and training it from scratch with the data available in this work.

Thus, one U-net was trained from scratch using the DAPI-Cells dataset to automatically segment mesenchyme cells in the sample, which are known to be drivers of the change in facial morphology. For this, only the mesenchyme cell annotations were used as the positive class in the training process, and the neural ectoderm cells and background were considered the negative class. Manual hyper-parameter search, including the learning rate and the number of iterations, was done using the ImageJ-Fiji plugin described above. The best model was trained for 12,500 iterations with a learning rate of 1E-4, with randomly initialized weights.

After this, a second U-net was trained from scratch based on the ImageJ-Fiji plugin using the PHH3-Cells dataset, for segmenting proliferating cells in pHH3 images. Based on a manual hyper-parameter search, the best model was trained for 20,000 iterations with a learning rate of 1E-6, with randomly initialized weights.

These U-net models were compared to the Ilastik segmentation method, which is a reference tool and widely used for semi-automatic cell segmentation in biology [17]. In this tool, the user specifies the image features to be extracted and the parameters of the machine learning method, and provides microscopy datasets with corresponding cell segmentations. Due to the number of tunable variables, its effectiveness depends on the domain expertise of the user. In case of LSM, it is challenging to optimize the tool so that it generalizes and performs well on future scans due to the variability between images and the large image size. The best results in this study were achieved using a random forest classifier integrating features based on intensity, edge, and texture. These features were computed using Gaussian-filtered images computed for sigmas of 3.5, 5, 10, and 15 pixels, independently for the DAPI-Cells and PHH3-Cells sets. Pixels with a score greater than 0.3 were assigned to the positive class.

Furthermore, the trained U-net models for cell segmentation were compared with Cellpose [27], a fully-automatic tool based on CNNs. Cellpose was trained with images of different cells, from a large variety of microscopy modalities, including DAPI-stained images. In this case, the tunable parameter *diameter* was tested with 0 (None), 12, 18, and 24, for the nuclei and its default models. For the DAPI-Cells set, the best results were obtained with the default model and zero diameter, which allows Cellpose to determine the optimal value. For the PHH3-Cells set, the best results were achieved with a diameter of 24 and the nuclei model.

### F. FULL VOLUME SEGMENTATION

Once the U-nets for segmentation of the tissues, cells, and proliferating cells are trained, full z-stacks can be segmented, and used to construct a full 3D model of the embryo.

For cell and tissue segmentation (Fig. 2) in an unseen LSM dataset, each image of the DAPI-stained z-stack has to be normalized and cropped to 1024 × 1024 patches so that it can be processed by the developed CNN models. Particularly for tissue segmentation, it was found experimentally that an overlap of neighbouring patches is necessary to avoid discontinuities of the segmentations at the borders of the patches. Once the segmentation of cells and tissues is completed and the patches are merged, the resulting anisotropic z-stacks of segmented images are converted to isotropic volumes, taking into account the in-slice resolution and spacing between slices of the original z-stack. Then, the mesenchyme segmentation is used to mask the cell segmentation volume so that only cells in mesenchyme remain in the segmentation.

For segmentation of proliferating cells, the images of the pHH3 z-stack also have to be intensity-normalized and cropped to 1024×1024 patches. These patches are segmented using the U-net trained with the PHH3-Cells dataset. Then, the segmented patches are merged, converted to isotropic volumes, and masked using the mesenchyme segmentation, to obtain the proliferating cells in the mesenchyme (Fig. 2).

The cell proliferation map is obtained by calculating the number of segmented voxels in a fixed region of interest corresponding to proliferating cells. The relative proliferation map is obtained by dividing the number of voxels of the proliferation map in a defined region of interest, with the corresponding number of voxels segmented as cells (proliferating and non-proliferating), in the same window.

### G. EVALUATION METRICS

At the pixel-level, the automatic segmentations were quantitatively compared to the corresponding manual segmentations in the three test sets using the accuracy, Dice similarity coefficient (DSC), recall, and precision as evaluation metrics, which are commonly used for assessing segmentation methods [19], [23], [31], [42], [43]. A global measure for assessing the tissue segmentation is required since it is a multi-class problem (mesenchyme, neural ectoderm, background). In this case, the macro-average F-score (Fscore_*M*_) was used, which is the average of the same measures for all classes [44]. As one aim of this work is to compute the cell proliferation and relative proliferation maps of embryos (Section II-F), these pixel-level metrics are used for quantitative analysis throughout the Paper.

Furthermore, object-level metrics of the cell segmentations were computed for complimentary analysis. This analysis is challenging due to the uncertain number of cells in each image, which applied to the ground-truth and automatic segmentation, considering the merging and/or splitting of objects (cells) that the automatic methods produce. In this work, the matching criterion used is that a cell in the ground-truth has a corresponding cell in the automatically extracted segmentation if there is an overlap of more than 50% between the segmented cells. A connected component analysis using a 4-connected neighborhood was performed to identify each cell from the annotations and automatic segmentations. The object-level F-score was used to quantify the agreement in object-level recognition [45], [46].^1^ The Hausdorff distance (HD, expressed in nanometers) was computed only for the cells in the ground-truth segmentations, which have matching cells in the automatically extracted segmentations.

## III. RESULTS

### A. TISSUE SEGMENTATION

As a computationally-intensive task, the hyper-parameter search for the tissue segmentation U-net CNN model was performed using the advanced research computing system Compute Canada, requesting an NVIDIA V100 Volta (32GB HBM2 memory). Using the identified best set of parameters for segmenting the validation set (Fscore_*M*_ = 0.804), a CNN model was trained using the training and validation set for segmenting the DAPI-Tissue test set using a PC running Ubuntu 18.04, with an AMD Ryzen 5 3600, 3.6 GHz 6-core CPU, 32 GB RAM, and an NVIDIA GeForce RTX 2070 Super GPU.

**FIGURE 4.**
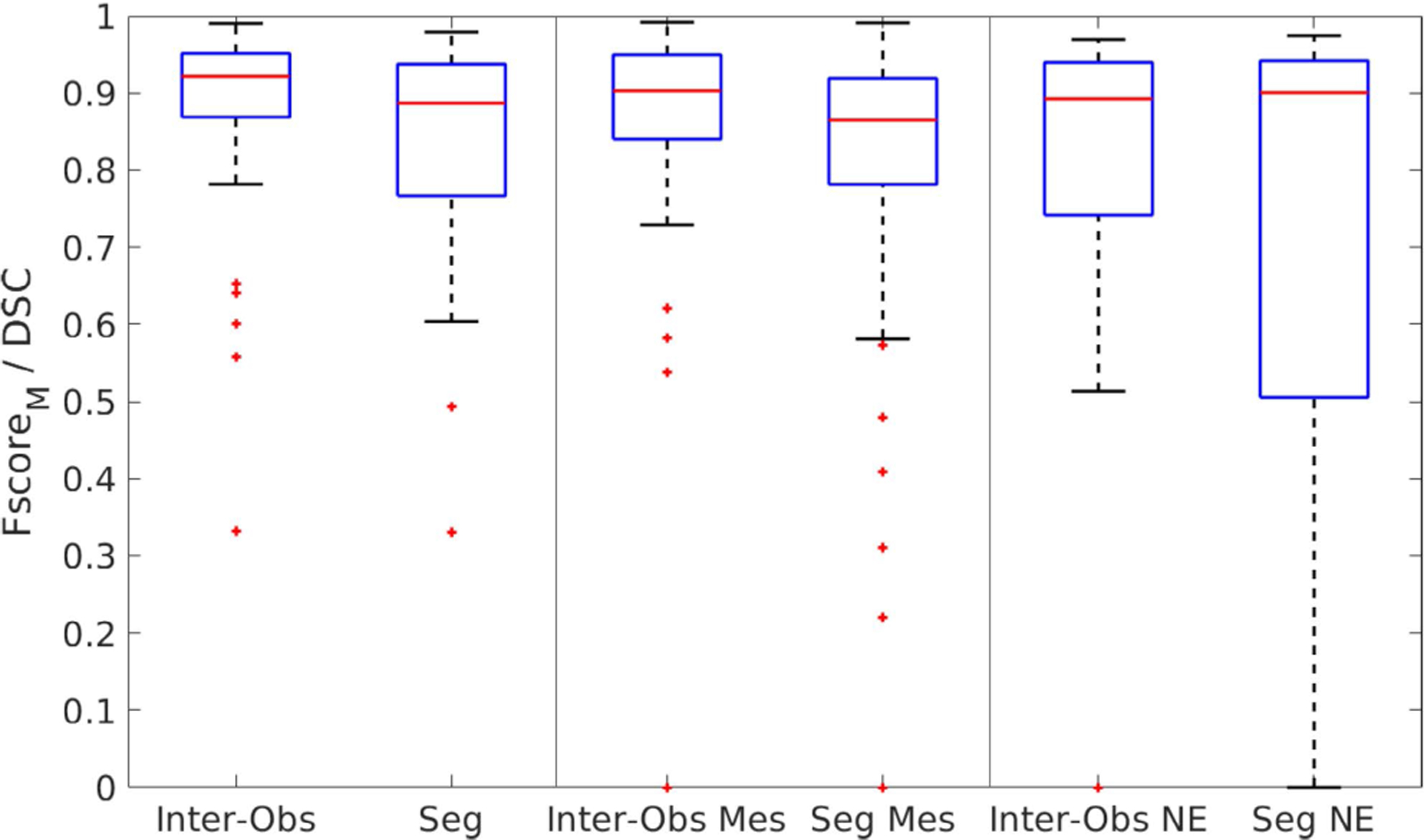
Box-plots of the overall Fscore_*M*_ and individual DSCs for neural ectoderm (NE) and mesenchyme (Mes) segmentation with the corresponding inter-observer agreement results for reference.

Table 2 shows the general metrics (accuracy, Fscore_*M*_) for the automatic segmentation of tissues for the validation and test sets. The DSC for background (DSC B) indicates the effectiveness of the developed CNN model to separate the sample from the background. Each metric (DSCs, accuracy) was computed for each image in the test and validation sets, and the global mean and standard deviation was computed for each set. As the CNN for tissue segmentation is trained from scratch, the metrics for the validation set are included to show that there is no overfitting to the training data. Table 3 shows the DSC, recall, and precision for each tissue of interest (mesenchyme and neural ectoderm) for the validation and test sets. Fig. 4 shows the box-plots for the metrics for the test set.

**FIGURE 5.**
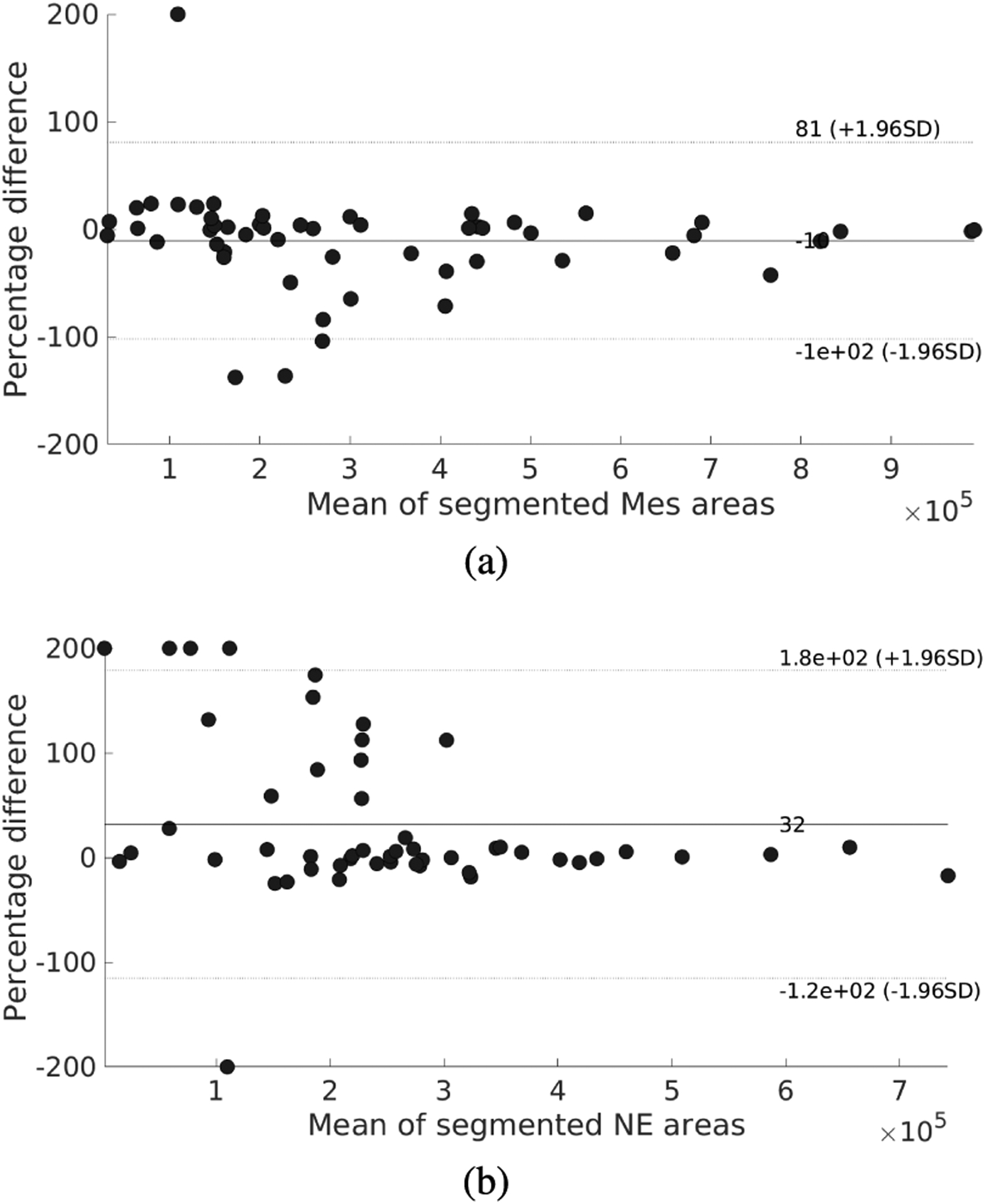
Bland-Altman plots for tissue segmentation. Areas are expressed in pixels. (a) Mesenchyme (Mes) segmentation. (b) Neural ectoderm (NE) segmentation.

Overall, the results show that the tissue segmentation using the proposed CNN model is similar to the manual segmentations. More precisely, a high accuracy of 0.889 was found for the test set, showing great agreement with the observers (Table 2). Considering the Fscore_*M*_, a metric more appropriate for multiclass imbalanced problems [44], the value slightly decreases to 0.837, which is lower than the Fscore_*M*_ of 0.885 for the inter-observer agreement. Based on a paired t-test, this difference is not statistically significant (*p* = 0.056 with *α* = 0.05). The mean DSCs for the mesenchyme and neural ectoderm segmentations (0.799 and 0.705, respectively) demonstrate high agreement with the observers, but slightly lower than the inter-observer variability (0.849 and 0.777, respectively). However, Fig. 4 reveals that the median DSCs for mesenchyme and neural ectoderm segmentations are 0.865 and 0.9, compared to 0.903 and 0.893 for the respective inter-observer values. These higher values for the DSCs show that the mean scores are affected by extreme low values, whereas the automatic segmentation highly agrees with the observers in most cases. Using paired t-tests, it was found that the difference between the mesenchyme and neural ectoderm segmentation distributions are not statistically significantly different to the distribution of the respective inter-observer values (*p* = 0.194 and *p* = 0.608, respectively, with *α* = 0.05). The analysis of the DSC for the background classification complements the understanding of the automatic segmentation performance (Table 2). In this case, a mean value of 0.931 is achieved, which is similar to the mean inter-observer agreement (0.916). The similar validation and test results show that the proposed model has a good generalization capability, not over- or under-fitting to the training data. Fig. 5a shows the Bland-Altman plots for the percentual error of the segmented areas, as the segmented areas vary between images. The horizontal distribution around 0% suggests that the error is equally distributed, indicating that no tissue is over- or under-estimated.

**FIGURE 6.**
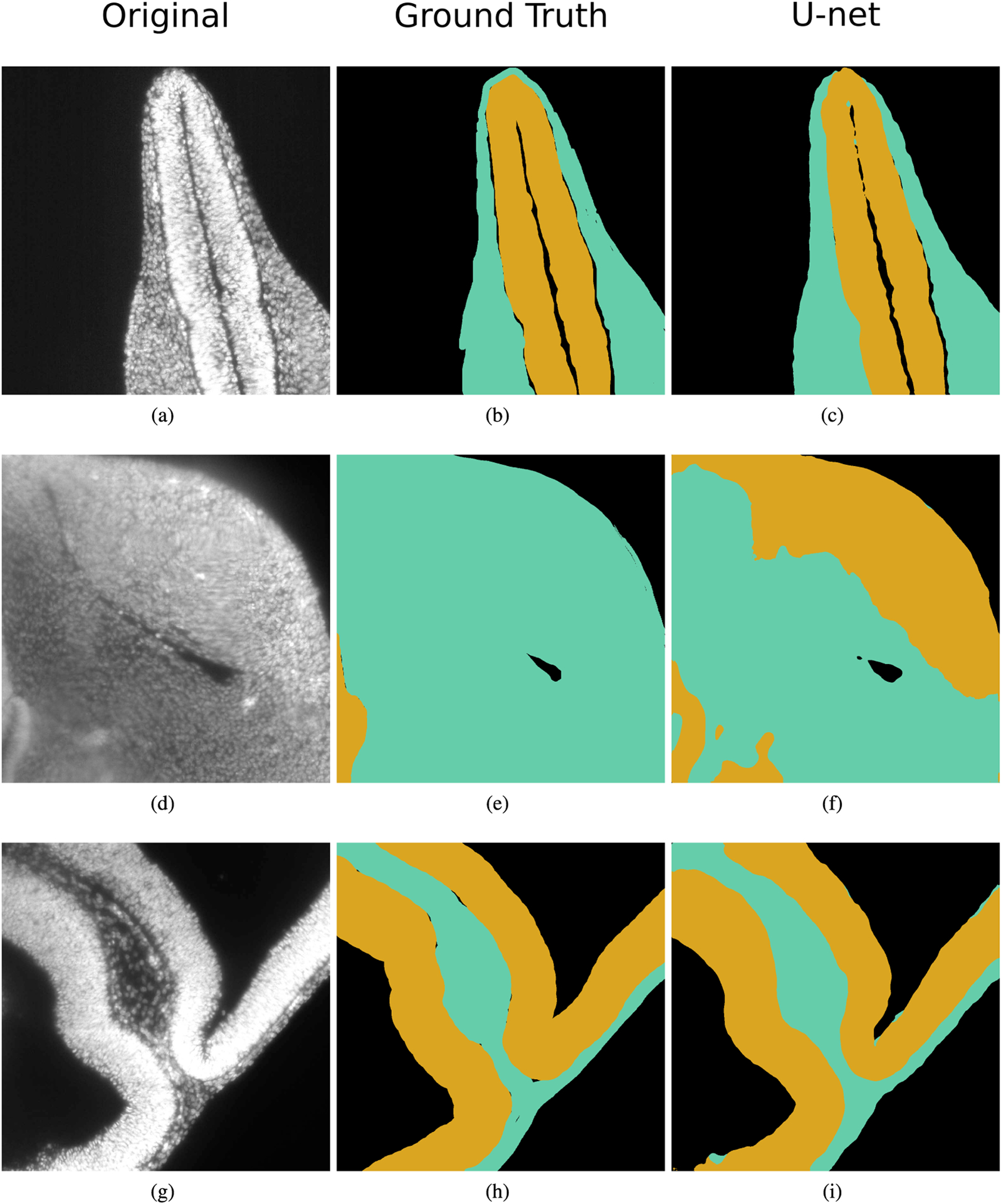
Segmentation of tissues in DAPI-stained images. (a) (d) (g) Original images from the DAPI-Tissue test set. (b) (e) (h) Ground truth, where the mesenchyme is coloured in aquamarine and the neural ectoderm in yellow. (c) (f) (i) Segmentation result using the proposed U-net for tissue segmentation.

Fig. 6 shows three selected images from the DAPI-Tissue test set with their corresponding manual and automatic segmentations: Fig. 6a shows one image without any significant image artifacts, which leads to good segmentation results. Fig. 6d displays an image with considerable intensity variations that result in the automatic method not performing as well as the manual observers. Finally, Fig. 6g shows an image with high surface curvatures of the tissues, which aside from some minor errors still results in overall good segmentation results.

### B. CELL SEGMENTATION

The fine-tuning of the U-net models for cell segmentation was performed on the same PC described above. The segmentation using Ilastik was executed on a PC running Ubuntu 18.04, with an Intel i7-8700K 3.7GHz 6-core CPU, 64 GB RAM, and a NVIDIA GeForce GTX 1080 Ti graphic card. The segmentation using Cellpose was performed using its console version, using a laptop with Windows 10, an Intel i7-8700H 2.20GHz, and 16 GB RAM.

Table 4 shows the evaluation metrics (accuracy, DSC, recall, precision, object-level F-score, and HD) for the automatic segmentation of mesenchyme cells in DAPI-stained images (DAPI-Cells, test set), including only images where the observer annotated at least one cell. Table 5 shows the results for the segmentation of proliferating cells in pHH3-stained images (PHH3-Cells, test set). In both tables, segmentation metrics for the validation set using the proposed U-net model are also provided to show that the training process is not overfitting the model.

**FIGURE 7.**
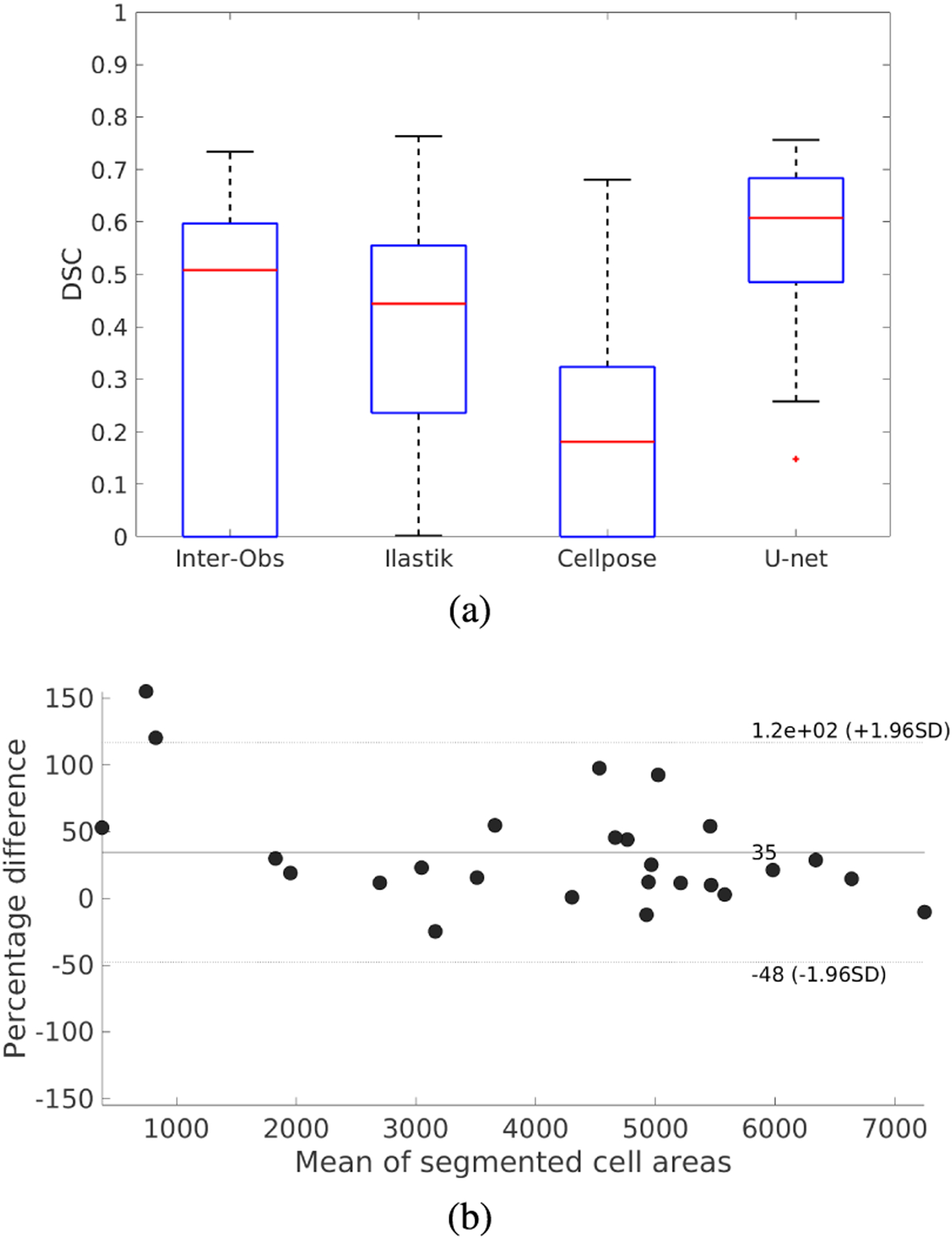
Box-plots of the Dice similarity coefficient (DSC) for cell segmentation in mesenchyme regions in DAPI-stained images using the DAPI-Cells test set. (a) DSCs for Ilastik, Cellpose, and U-net, compared to the inter-observer agreement. (b) Bland-Altman plot for the proposed U-net segmentation method. Areas are expressed in pixels.

**FIGURE 8.**
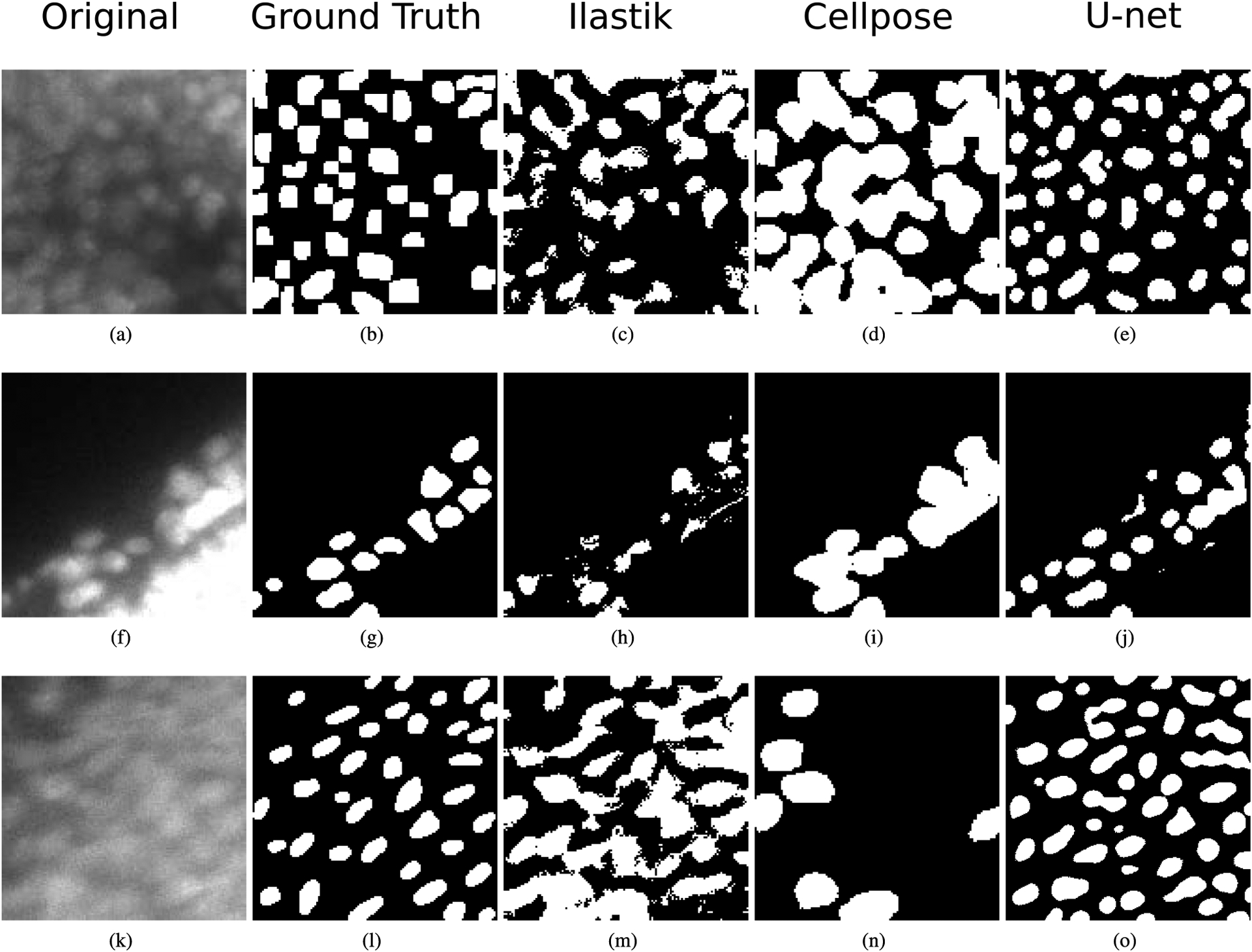
Segmentation of cells in DAPI-stained images. (a) (f) (k) Original images from the DAPI-Cells test set. (b) (g) (l) Ground truth in which only mesenchyme cells are segmented. (c) (h) (m) Segmentation result using Ilastik. (d) (i) (n) Segmentation result using Cellpose. (e) (j) (o) Segmentation result using the proposed U-net.

For the mesenchyme cell segmentation in DAPI images, Fig. 7 presents the box-plots for the DSCs when segmenting the test set of DAPI-Cells, and the corresponding Bland-Altman plot for the percentual error of the segmented areas. Table 4 and Fig. 7a show that the U-net model achieves a DSC of 0.569, which is considerable better than the Ilastik and Cellpose results (0.399 and 0.203, respectively). Fig. 7b shows that the segmentation error is equally distributed around 0%, indicating no systematic bias. Qualitatively, Fig. 8 shows segmentation examples in mesenchyme regions. Here, due to the high density of cells, individual nuclei do not have clear borders. Also, their intensities can vary among the images due to their position in the sample and the amount of light received in their region, and variations in the staining and clearing process. Comparing the three automatic segmentation methods, it becomes apparent that the proposed U-net model produces sharper segmentations than Ilastik, looking more similar to the ground truth. Cellpose produces sharp segmentations, but tends to merge cells, not identifying their blurry edges in the images (Fig. 8d and 8i). The superior segmentation sharpness produced by the U-net is quantitatively evidenced by the lowest Hausdorff distance found for the automatic segmentation methods (9.69 nm). Thus, the proposed U-net is the only method that is able to segment cells from the high-density and overexposed regions leading to results in the range of the inter-observer agreement (Fig. 8j).

**FIGURE 9.**
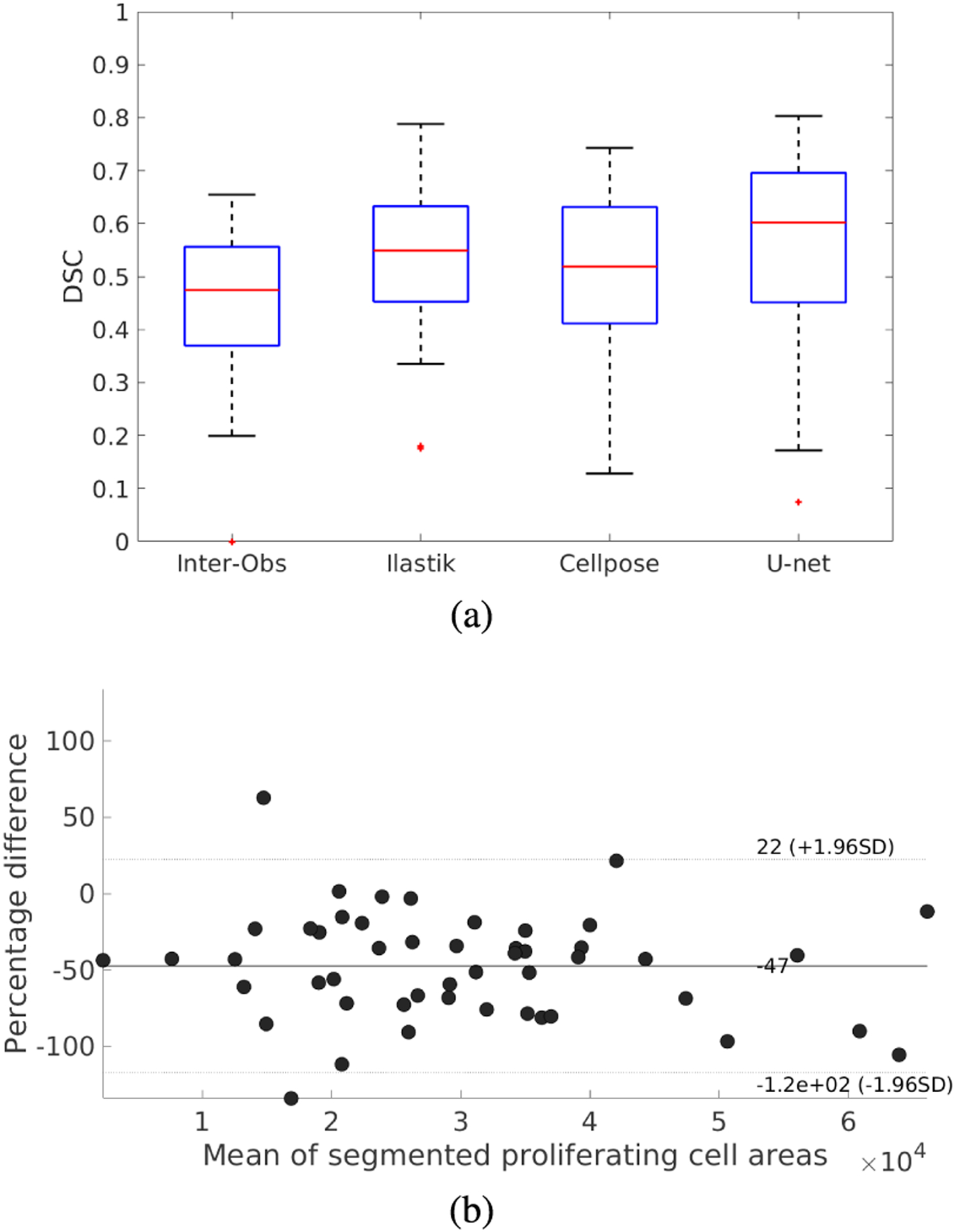
Box-plots of the Dice similarity coefficient (DSC) for proliferating cell segmentation in pHH3 images. (a) DSCs for Ilastik, Cellpose, and U-net compared to the inter-observer agreement. (b) Bland-Altman plot for the proposed U-net segmentation method. Areas are expressed in pixels.

**FIGURE 10.**
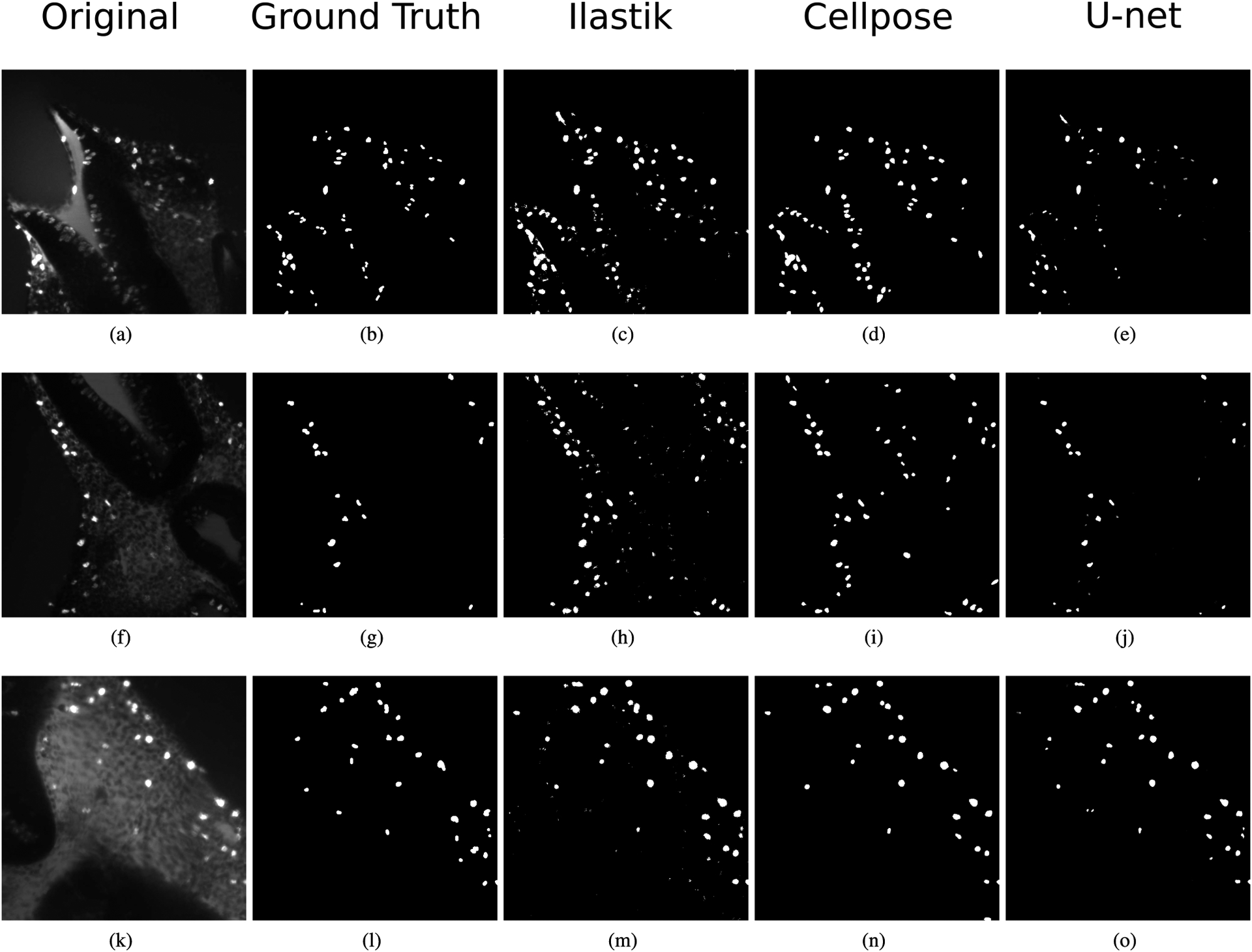
pHH3-stained image segmentation. (a) (f) (k) Sections of the original images from the PHH3-Cells test set. (b) (g) (l) Ground truth. (c) (h) (m) Segmentation result using Ilastik. (d) (i) (n) Segmentation result using Cellpose. (e) (j) (o) Segmentation result using the proposed U-net.

For the PHH3-Cells segmentation, the proposed U-net achieved a DSC of 0.56 for the test set, compared to the lower mean inter-observer agreement DSC of 0.452 (Table 5). The performance of the proposed U-net is slightly better than Ilastik (0.537) and Cellpose (0.503). In terms of the object-level F-score, the CNN-based methods (Cellpose and U-net) have similar performance (0.537 and 0.524, respectively) and perform better than Ilastik. Fig. 9 shows the box-plots for the DSCs obtained on the PHH3-Cells test set and the associated Bland-Altman plot for the U-net segmentation. Fig. 9b indicates a tendency towards under-estimation for the U-net method. Fig. 10 shows examples of the segmentation of proliferating cells. The images selected show different levels of background noise, which directly affect the segmentation results. It can be seen that Ilastik does not produce segmentations as sharp as the CNN-based methods. Furthermore, Cellpose and Ilastik over-estimate the presence of proliferating cells in comparison with the U-net model, as the quantitative results presented in Table 5 suggest: Ilastik and Cellpose have higher recall and lower precision values than the U-net, which favours precision instead of recall. Finally, the U-net achieved the best HD value (4.31 nm) in comparison with Ilastik and Cellpose (6.32 and 6.03 nm, respectively). Thus, for the dataset in this work, U-net is the best method for segmenting proliferating cells in pHH3-stained images among the tested methods.

### C. WORKFLOW INTEGRATION

**FIGURE 11.**
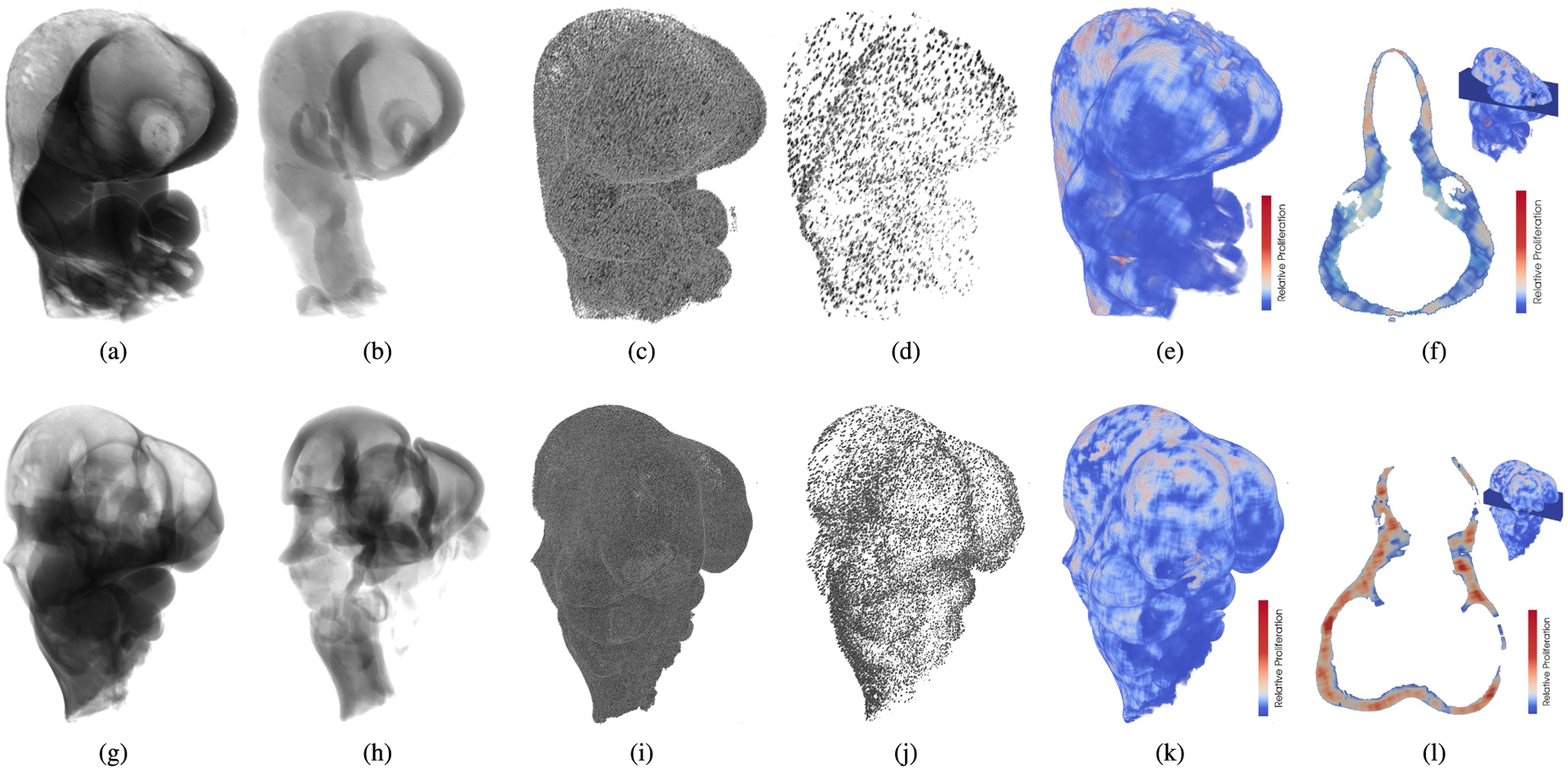
Automatically segmented E9.5 (top) and E10.5 (bottom) mouse embryos. For visualization purposes, the volume scales are not preserved, and voxels are displayed slightly transparent. (a) (g) Mesenchyme tissue segmented from DAPI images. (b) (h) Neural ectoderm tissue segmented from DAPI images. (c) (i) Cells in the mesenchyme segmented from DAPIstained images. (d) (j) Proliferating cells segmented in pHH3-stained images, only in the mesenchyme, masked using the tissue segmentation result. (e) (k) Heat map of the relative proliferation in the mesenchyme. (f) (l) Axial plane located in the head of the embryo, showing the relative proliferation in the mesenchyme, after masking out the neural ectoderm.

The trained U-nets for segmentation of the tissues, cells, and proliferating cells enable the processing of z-stacks of whole embryo scans. One E9.5 and one E10.5 mouse embryo head were segmented and are shown in Fig. 11. For this, an overlap of the patches by 100 pixels on each side was used for tissue segmentation. The resulting segmented slices (tissues, cells, and proliferating cells) were downsampled to generate isotropic volumes to account for the higher in-slice resolution of LSM images compared to the slice thickness.

Qualitatively, the volumes of the segmented neural ectoderms (Fig. 11b and Fig. 11h) have the expected shape for the embryonic ages and fit within the segmented mesenchyme (Fig. 11a and 11g), as they should. The total number of segmented cells in the scan displayed in Fig. 11c and Fig. 11i is higher than the total number of proliferating cells, which can be clearly seen in Fig. 11d and Fig. 11j, respectively. Using these numbers, the relative proliferation map is computed (Fig. 11e and Fig. 11k). For example, it can be seen in Fig. 11k that the frontal nasal prominences show higher rates of proliferation than in the previous stage (Fig. 11e). Fig. 11f and Fig. 11l show the proliferation in the mesenchyme, and an empty space corresponding to the neural ectoderm, which has been masked out.

## IV. DISCUSSION

### A. TISSUE SEGMENTATION

The results of this study suggest that the proposed CNN-based framework is able to segment the background as well as a human observer, but it can lead to some misclassifications of the mesenchyme and neural ectoderm. In Fig. 6, it can be seen that the embryo is well separated from the background, but the tissues are partly mislabeled. For example, in Fig. 6f, mislabelling occurs in blurry regions of the image (bottom left) and where the signal varies significantly (top right). In this case, these artifacts in the image are a result of the staining process and LSM acquisition artifacts. However, the proposed CNN-based segmentation method still leads to good results in some of these regions with less severe artifacts.

Overall, the good performance of the automatic tissue segmentation enables the use of subsequent geometric morphometrics analysis, which is a standard for developmental biology research.

### B. CELL SEGMENTATION

With respect to the cell segmentation, the results show a rather low inter-observer agreement with a DSC of 0.39 for cell segmentation in DAPI-stained images and 0.45 for cell segmentation in pHH3-stained images. Furthermore, the U-net segmentation achieves mean DSCs of 0.57 and 0.56, respectively, statistically significantly higher than the inter-observer agreement (Tables 4 and 5). This low agreement has also been reported for other fluorescence microscopy datasets [42], and is likely related to the differences in cell density across the tissues, where some areas have very high cell densities and cells appear superimposed on each other or with blurry cell edges even in regions with optimal focus. Also, it is worth noting that the staining and optical clearing were performed on whole embryos in this work, which makes sample preparation more difficult compared to the preparation of typical physical slices [18], [21], [47].

In order to assess the extent to which the quality of the scans affects the human and automatic segmentation, the image focus assessment method proposed by Yang *et al*. [14] was used. In DAPI-Cells test images with low defocus scores (between 1 and 5), the DSC comparing the manual segmentations was 0.49±0.22, whereas comparing the automatic segmentation results with the manual segmentation results led to a DSC of 0.63±0.1. For images with high defocus scores (between 6 and 11), the inter-observer DSC is 0.26±0.27, whereas the corresponding value for the automatic segmentation is 0.47±0.17. Thus, the loss of focus affects the manual segmentation much more than the automatic segmentation. It can be concluded that the low effectiveness of the automatic segmentation is strongly related to the quality of the training data, which includes the image quality but also the manual segmentations used. Furthermore, this shows that an automatic assessment of image quality would be useful before incorporating images and/or samples into a larger study to reduce noise in the data.

The DSC, accuracy and HD values reached by the U-net method for cell segmentation are better than the corresponding metrics for the Ilastik and Cellpose segmentations for both DAPI and pHH3 images (Tables 4 and 5). Furthermore, the CNN-based methods (U-net and Cellpose) produce better defined cells, compared to the Ilastik results (Fig. 8 and 10), which is likely a consequence of taking high level textural features into account, which are computed in deeper CNN layers. These features are not available or computed in the Ilastik method, even when it is configured by users trained in image processing. In case of PHH3-Cells, it can be argued that the acceptable performance of the three segmentation techniques is a result of the lower number of cells that bind the staining in comparison with the DAPI stain, resulting in images with lower density of objects of interest and sharper edges.

The CNN-based methods perform similarly for the PHH3-Cells set, both quantitatively (Fig. 9a) and qualitatively (Fig. 10), but not for DAPI-Cells (Table 4). Cellpose was trained with a large and diverse set of image datasets that enhance its performance and generalization capability. However, the DAPI (all nuclei stained) images acquired and used in this work (Section II-A) present a myriad of challenges, which are distinct from other microscopy methods and is likely the reason for the worse performance found. More precisely, the images generated using LSM have a number of artifacts resulting from the clearing and staining processes. Most importantly, the LSM datasets show some optical aberrations as the light-sheet passes through the tissues. This is due to slight differences in clearing and any abnormalities in the embedding media (agarose plug). These aberrations create blurred areas in the specimens that must be identified to properly analyse the data. Furthermore, some regions of the tissue have much higher cell densities than others, making it difficult to apply uniform methods (Fig. 8f). Most microscopy methods for imaging of dense/opaque tissues require sectioning and methods developed for those types of images are likely to have higher quality and fewer artifacts than the data used in this work. Thus, it was necessary to train a CNN-based method (U-net) to obtain competitive results using LSM datasets. It is worth noting that only the ImageJ-Fiji plugin [24] was tested for transfer learning in this work, without performance advantages. However, using other pre-trained networks such as Cellpose and the one described by Schimdt *et al*. [48] might lead to quantitative advantages. In addition to that, other CNN architectures such as ResNet [49] or SegNet [50] might also lead to better results. However, given that the U-net was developed specifically for cell segmentation, performs well for many segmentation problems, and achieves results in the range of the inter-observer agreement may suggest that other networks may not lead to a significant improvement.

### C. DATASETS

The annotated and freely available DAPI-Cells and PHH3-Cells datasets can be used to develop more advanced cell segmentation methods. Within this context, it is well known that the amount of data can be very important for the training of deep learning models [51]. Considering the current body of literature, the number of images available for training and evaluation in this study is rather large. While adding more images may improve the accuracy of deep learning models to some extent, the rather small difference between the validation and test metrics observed in this work as well as the fact that the results are similar to the inter-observer agreement suggest that the potential for improvement by adding more datasets may be limited.

Much rather, the low inter-observer agreement and the high blurriness of the original images suggest that more research is needed to improve the preparation of samples, the LSM imaging setup to reduce artifacts directly, or the image processing methods, including the deep learning models. This will likely lead to better segmentation results than simply adding more images to the database.

### D. WORKFLOW INTEGRATION

Fig. 11 shows each segmentation result as generated by the proposed workflow in E9.5 and E10.5 embryo heads. All segmentations fulfill the expected properties. However, some unexpected sagittal asymmetries are present. They can be seen in the resulting mesenchyme shape and in the distribution of the relative proliferation in Fig. 11f and Fig. 11l. These asymmetries could be the result of variance in the staining of the embryo, in both DAPI and pHH3, sample orientation during scanning, the laser penetration, and errors in the automatic segmentation methods. Asymmetries could affect further shape and morphological analysis. As a sagittal symmetry of their morphology and proliferation can be assumed in wild-type mice, these asymmetries can be corrected in the segmentations using standard post-processing methods such as affine deformations based on an embryo atlas or predefined landmarks.

Generally, the overall results show that the CNN models can segment the structures of interest accurately and in the range of human observers. Thus, this automatic segmentation workflow can be efficiently used for quantitative analysis of LSM images oriented to developmental biology studies. However, the inter-observer metrics show that there is room for improvement with respect to the quality of the image acquisition.

## V. CONCLUSION

Proper tissue and cell segmentation is important for the quantification and modelling of how perturbation to cellular dynamics results in congenital anomalies in mouse models. Such analyses are based on data that rely heavily on the extraction of variables such as cell number, size, or density from noisy volumetric image data. The proposed pipeline using the well-established U-net CNN architecture leads to segmentation results within the range of the inter-observer agreement and can therefore help to accelerate LSM image analysis and generate reproducible results. Moreover, the proposed CNN-based segmentation framework together with the annotated datasets can serve as a reference for comparison of more advanced LSM image segmentation methods in future. Furthermore, it should be highlighted that the proposed cell segmentation approach can be easily extended to additional punctate stains other than DAPI and phospho-Histone H3.

The source code, software, instructional videos, and annotated datasets are publicly available at https://github.com/lucaslovercio/LSMprocessing.

## Figures and Tables

**TABLE 1.** Distribution of the images for training (train), validation (val) and test, sizes and annotated classes for the three proposed datasets.

Dataset	Train	Val	Test	Size	Classes
DAPI-Tissue	86	36	54	1024 × 1024	(1) Mesenchyme (2) Neural ectoderm (3) Background
DAPI-Cells	96	17	55	131 × 131	(1) Mesenchyme cell (2) Neural ectoderm cell (3) No cell
PHH3-Cells	86	20	49	1024 × 1024	(1) Proliferating cell (2) No proliferating cell

**TABLE 2.** Mean and standard deviation of the metrics for the segmentation of tissues and background (B) in DAPI-Tissue images using the proposed U-net. p-values were computed using a paired t-test, where the null hypothesis states that the mean difference between pairs of manual and automatic segmentations is zero.

	Accuracy	Fscore_*M*_	DSC B
Inter-observer (Test)	0.971(0.031)	0.885(0.122)	0.916(0.146)
U-net (Val)	0.900(0.080)	0.804(0.133)	0.974(0.015)
U-net (Test)	0.889(0.093)	0.837(0.137)	0.931(0.141)
	*p <* 0.001	*p* = 0.056	*p* = 0.58

**TABLE 3.** Mean and standard deviation of the metrics for the segmentation of mesenchyme (Mes) and neural ectoderm (NE) in DAPI-Tissue images using the proposed U-net. p-values were computed using a paired t-test, where the null hypothesis states that the mean difference between pairs of manual and automatic segmentations is zero.

	DSC Mes	Recall Mes	Precision Mes	DSC NE	Recall NE	Precision NE
Inter-observer (Test)	0.849(0.196)	0.855(0.209)	0.864(0.204)	0.777(0.261)	0.755(0.327)	0.739(0.322)
U-net (Val)	0.783(0.208)	0.919(0.164)	0.711(0.232)	0.515(0.323)	0.417(0.282)	0.741(0.403)
U-net (Test)	0.799(0.201)	0.777(0.238)	0.866(0.162)	0.705(0.337)	0.798(0.299)	0.688(0.363)
	*p* = 0.194	-	-	*p* = 0.608	-	-

**TABLE 4.** Mean and standard deviation of the metrics for mesenchyme cell segmentation in DAPI-stained images (DAPI-Cells), using Ilastik, Cellpose, and the trained U-net. Best results for the test set are highlighted in bold. p-values were computed using a paired t-test, where the null hypothesis states that the mean difference between pairs of manual and automatic segmentations is zero.

	Accuracy	DSC	Recall	Precision	F-score	HD (nm)
Inter-observer (Test)	0.811(0.096)	0.385(0.279)	0.404(0.299)	0.409(0.298)	0.362(0.279)	11.454(10.851)
Ilastik (Test)	0.797(0.097)	0.399(0.209)	0.409(0.257)	**0.515(0.179)**	**0.426(0.167)**	26.704(21.708)
	*p* = 0.633	*p* = 0.831	-	-	-	-
Cellpose (Test)	0.789(0.106)	0.203(0.201)	0.183(0.247)	0.349(0.266)	0.148(0.156)	18.096(12.514)
	*p* = 0.453	*p* = 0096	-	-	-	-
U-net (Val)	0.811(0.078)	0.649(0.154)	0.825(0.076)	0.558(0.183)	-	-
U-net (Test)	**0.812(0.081)**	**0.569(0.156)**	**0.691(0.107)**	0.513(0.179)	0.217(0.114)	**9.688(6.742)**
	*p* = 0.977	*p* = 0.005	-	-	-	-

**TABLE 5.** Mean and standard deviation of the metrics for segmentation of proliferating cells in pHH3-stained images (PHH3-Cells), using Ilastik, Cellpose, and the trained U-net. Best results for the test set are highlighted in bold. p-values were computed using a the paired t-test, where the null hypothesis states that the mean difference between pairs of manual and automatic segmentations is zero.

	Accuracy	DSC	Recall	Precision	F-score	HD (nm)
Inter-observer (Test)	0.987(0.006)	0.452(0.133)	0.467(0.175)	0.745(0.181)	0.485(0.165)	8.059(14.034)
Ilastik (Test)	0.989(0.005)	0.537(0.134)	**0.763(0.209)**	0.445(0.143)	0.323(0.123)	6.317(2.349)
	*p* = 0.102	*p* = 0.002	-	-	-	-
Cellpose (Test)	0.989(0.005)	0.503(0.157)	0.719(0.233)	0.432(0.172)	**0.537(0.171)**	6.029(1.577)
	*p* = 0.293	*p* = 0.086	-	-	-	-
U-net (Val)	0.989(0.006)	0.471(0.128)	0.346(0.129)	0.809(0.059)	-	-
U-net (Test)	**0.994(0.004)**	**0.560(0.172)**	0.468(0.176)	**0.745(0.181)**	0.524(0.139)	**4.309(1.218)**
	*p* < 0.001	*p* < 0.001	-	-	-	-

## Footnotes

1 DSC and F-score have the same mathematical formulation. However, DSC is usually used for pixel-level analysis, while the F-score is associated with binary classification of objects.
